# Assessing courtesy reporting bias in facility-based surveys on person-centred maternity care: evidence from urban informal settlements in Nairobi and Lusaka

**DOI:** 10.7189/jogh.15.04090

**Published:** 2025-03-28

**Authors:** Safia S Jiwani, Martin Kavao Mutua, Choolwe Jacobs, Mwiche Musukuma, Anne Njeri, Godfrey Adero, Dennis Ngosa, Amanuel Abajobir, Cheikh Mbacké Faye, Ties Boerma, Agbessi Amouzou

**Affiliations:** 1 Department of International Health, Johns Hopkins Bloomberg School of Public Health, Baltimore, Maryland, USA; 2 African Population and Health Research Centre, Nairobi, Kenya; 3 Department of Epidemiology and Biostatistics, School of Public Health, University of Zambia, Lusaka, Zambia; 4 Centre for Infectious Disease Research in Zambia, Lusaka, Zambia; 5 African Population and Health Research Centre, Dakar, Senegal; 6 Population and Global Health, University of Manitoba, Winnipeg, Manitoba, Canada

## Abstract

**Background:**

Experience of care is typically measured through client exit surveys administered in the facility. Evidence suggests that such measures suffer from courtesy reporting bias whereby respondents do not accurately report on their experiences while in the facility. We explored the presence of courtesy bias by comparing women’s reported experience of person-centred maternity care (PCMC) from facility-based client exit surveys to mobile phone-based surveys out of the facility in Nairobi and Lusaka’s urban informal settlements.

**Methods:**

We randomly and independently sampled women in the facilities for either a facility-based survey (n = 233 in Lusaka and n = 112 in Nairobi) or a mobile phone-based survey (n = 203 in Lusaka and n = 300 in Nairobi) within one to two weeks of facility discharge. The questionnaire included a validated PCMC scale. After adjusting for differences in women’s characteristics across groups, we compared PCMC scores between facility and phone-based samples. We ran multilevel linear regression models to assess PCMC by survey modality in each city.

**Results:**

In both cities, over 70.0% of women were aged 20–34 years and were married, at least two thirds had secondary education, and over 95.0% were unaccompanied during labour/delivery. The overall PCMC score was 69.3% among women surveyed on the phone compared to 70.2% among those surveyed in the facility in Nairobi. In Lusaka, it was 57.5% on the phone compared to 56.8% in-facility. We found no statistically significant differences in PCMC scores between survey modalities in both cities, after adjusting for differences in women’s characteristics.

**Conclusions:**

We did not detect significant courtesy reporting bias in PCMC in facility-based client exit surveys in the context of urban informal settlements in Nairobi and Lusaka. Experience of PCMC can be measured through in-facility client exit surveys or mobile phone surveys. However, it is critical to address challenges related to a mobile phone-based approach.

A staggering 70% of the world’s maternal deaths in 2020 occurred in sub-Saharan Africa [1]. In parallel, the high rural-to-urban migration over the last decade has led to an uncontrolled growth of cities in this region, where the poorest populations too often get trapped in precarious living conditions and face an unmet need for high-quality care [2,3]. The majority of maternal and neonatal deaths can be prevented by ensuring access to high quality health services during pregnancy, childbirth and the postpartum period [2,4].

Quality of care measurement encompasses three core dimensions, based on the World Health Organization framework for monitoring maternal and newborn health, and building off the Donabedian model – provision of care, user experience of care, and facility readiness for high quality services [5,6]. Most studies evaluating quality of maternal and newborn care rely on women’s self-report and recall of interventions received. Person-centred maternity care (PCMC) is a measure of experience of care, defined as maternity care that is respectful of and responsive to women’s and their families’ preferences, needs and values, and ensuring that their values guide clinical decisions [7]. Afulani et al. developed and validated a 30-item PCMC scale covering domains of patient dignity and respect, communication and autonomy, and supportive care [8,9]. While direct observations of care remain the gold standard for measuring provision and experience of care, this method is resource and time intensive, leading researchers to rely on client exit surveys typically conducted in health facilities.

Several authors have expressed concern about the presence of courtesy reporting bias in client exit surveys, particularly when patients are surveyed in the facility upon receiving health services. Courtesy reporting bias may occur when patients refrain from freely sharing their true experience of care or from reporting negative experiences while they are still in the facility and surrounded by providers [10–13]. They may confuse the research team as staff of the facility, thus biasing their responses [14]. Courtesy reporting bias may lead to the under-reporting of negative experiences or disrespect and abuse, over-reporting of satisfaction with services [10–13,15], and compromises the reliability of such measures [14]. This is a concern in low- and middle-income countries, and in particular among low-income populations relying on free maternity care services during pregnancy and the postpartum period. Women may be unwilling to share their true experiences out of fear of offending health providers, losing access to services or facing repercussions; it is therefore relevant in the context of urban informal settlements. Courtesy reporting bias has largely affected subjective and sensitive questions assessing experience and satisfaction of care, interpersonal patient-provider relationship, and reporting of disrespectful care [11,16–18]. A systematic review of methods used to quantify disrespect and abuse during childbirth found that courtesy bias was a common challenge in accurately estimating the prevalence of disrespect and abuse, and hindered comparisons across studies with varying data collection settings. The authors recommended conducting client exit interviews in a neutral, safe place rather than in the facility or its vicinity [15].

In this study, we assessed the presence of courtesy reporting bias in facility-based client exit surveys measuring the experience of PCMC among women living in urban informal settlements in Nairobi and Lusaka. We did this by comparing estimates of self-reported PCMC between in-person facility-based client exit surveys and mobile phone-based surveys administered days following facility discharge. We assumed that women surveyed on the phone (out of the facility) were not affected by courtesy reporting bias. Based on previous evidence [10–13], we hypothesised that women surveyed in the facility were less likely to accurately report negative experiences of care and would therefore have higher PCMC scores than those surveyed on the phone.

## METHODS

### Study design and sampling

We conducted this analysis within a larger study evaluating the experience and determinants of PCMC among women living in urban informal settlements in sub-Saharan African capital cities. The cross-sectional study is based on client exit surveys of a random sample of women of reproductive age (15–49 years) living in informal settlements who were discharged from childbirth care in health facilities identified as serving the study area, consisting of select urban informal settlements in Nairobi and Lusaka cities. The client exit survey was implemented using two distinct survey modalities: an in-person survey held in the facility, or a mobile phone-based survey administered within one to two weeks of facility discharge. Importantly, PCMC questions between the two survey modalities in a given city were identical, and the two samples were drawn from the same health facilities.

In each city, we calculated a precision-based sample size of 418 women to estimate PCMC with a three percentage-point (pp) margin of error, based on the literature and affordability. This overall sample was split between phone-based and facility-based samples drawn randomly and independently of each other. Power analyses revealed that our sample sizes allowed detecting differences of 4.2 pp and 3.6 pp in PCMC scores between the two groups in Nairobi and Lusaka respectively, with 80% power. Recruitment was conducted sequentially: we first recruited women at the facility for the mobile phone-based sample and followed-up with a phone call within one to two weeks of delivery discharge. Once we had achieved the desired sample of phone-based surveys, we recruited women in the facility for the in-person surveys. All women, regardless of the survey modality, were recruited in the facility upon assessing their eligibility using a screening form. Eligible and interested participants provided written consent in the facility after which the in-person survey was administered, or an appointment was made for the phone-based survey at a time convenient for the participant. Further details on the study design, setting, sample size calculation and data collection have been described elsewhere [19].

In Nairobi, we conducted 300 phone-based and 112 in-person surveys at the facility upon discharge. In Lusaka, we conducted 203 phone-based and 233 in-person surveys. Given higher delivery volumes and challenges in approaching women immediately after delivery, in Lusaka, in-person client exit surveys were held at postnatal facilities one to two weeks following delivery discharge. All in-person surveys were conducted in an area of maximum auditory and visual privacy in the facility. For the phone-based survey sample, our study team conducted three attempts to reach women, after which a text message was sent to assess their availability. When a suitable time was identified, we ensured women felt comfortable speaking on the phone in an area of maximum privacy. We gauged for possible distractions and if necessary, we rescheduled the interview to ensure their availability and attention.

### Measures

We estimated PCMC following the methodology developed by Afulani et al., whereby a score ranging from zero to three was assigned to each of the 30 items making up the PCMC scale (Table S1 in the **Online Supplementary Document**) [8,9]. Responses were on a four-point scale (no never, yes a few times, yes most of the time, yes all the time) and the highest numeric value of three was attributed to the response reflecting person-centred care. Values for all 30 items were then summed, for a maximum score of 90 points for the full PCMC scale; a higher score therefore reflected higher PCMC. We also generated domain-specific scores for each of the three domain subscales: dignity and respect (six items for a total of 18 points), communication and autonomy (nine items for a total of 27 points), and supportive care (15 items for a total of 45 points). For ease of interpretation as a percentage, we rescaled overall and domain scores to 100.

### Statistical analysis

We assessed normality of the unscaled PCMC score by plotting a histogram of the scores as well as quantile-quantile plots of the residuals (Figure S1 in the **Online Supplementary Document**). We compared the rescaled overall and domain-specific PCMC scores with corresponding 95% confidence intervals (CIs) between facility-based and phone-based samples using two-sample *t* tests. We then ran a two-level random intercept linear regression model of unscaled PCMC scores (a continuous outcome) on the survey modality (a dichotomous predictor), for each city separately. Level one referred to individual women and level two referred to the facilities they attended, thus accounting for clustering of women within facilities and variability in PCMC scores across facilities.

While we sampled women randomly and independently for the facility and phone-based samples in each city, there may have been differences in the composition of women’s characteristics across groups. Our previous analyses identified key structural, intermediary and health systems factors associated with women’s PCMC reporting in this setting [19]. We therefore compared the phone and facility samples for such differences, including: women’s age, education, marital status, employment, parity, history of miscarriage/stillbirth or caesarean section, pregnancy complications, care seeking for antenatal care (ANC), receipt of maternal and newborn postnatal care (PNC) before discharge, as well as delivery facility type, managing authority, type of provider assisting during labour and delivery, delivery complications, length of stay in the facility, and overall satisfaction with childbirth care. To be conservative, we adjusted the models by characteristics that were statistically significantly different across groups based on a chi square *P* value <0.2. In Lusaka, we adjusted the models by women’s age, employment, parity, number of ANC contacts, whether they had a private bed for labour/delivery, the length of facility stay for delivery, and whether a newborn PNC appointment was received prior to discharge. In Nairobi, the models were adjusted by women’s age, marital status, parity, history of a miscarriage/stillbirth, any pregnancy complications, decision to delivery in a facility, whether they had a private bed for labour/delivery, and whether a newborn check was conducted prior to discharge.

As a supplementary subgroup analysis, we compared mean PCMC scores by women’s characteristics, between facility-based and phone-based samples in each city, to assess courtesy reporting bias within subgroups of women (Table S2 in the **Online Supplementary Document**).

## RESULTS

### Sample characteristics

The response rates varied by survey modality, with lower responses in the phone-sample in both cities. Challenges with reaching women over the phone and finding a suitable time for the interview led to larger loss to follow-up. The response rate for phone surveys in Lusaka was 81.4% compared to 95.5% in-facility, and in Nairobi it was 91.1% compared to 100% in-facility.

In Lusaka, most women were aged 20–34 years, though they made up 71.7% of the facility-based sample compared to 83.3% of the phone-based sample. The facility-based sample had a larger proportion of adolescents aged 15–19 years (14.6% in-facility compared to 6.9% in the phone sample) and older adults aged 35–49 years (13.7% in-facility compared to 9.9% in the phone sample). Similarly, a larger proportion of the facility-based sample was primiparous, though most women had two to three children. More than half of the women across samples were unemployed (63.1% in-facility compared to 51.2% in phone sample). While care seeking for ANC was high in both groups, fewer women in the facility sample had never sought ANC. In contrast, fewer women in the facility sample reported receiving a newborn PNC appointment before discharge (93.6% in facility *vs.* 99.0% on the phone). In Nairobi, a smaller proportion of women in the facility sample were married/in union (81.2% in-facility compared to 89.3% on the phone), and a larger proportion reported receiving a newborn PNC check prior to discharge (98.2% in-facility compared to 92.3% on the phone) (**Table 1**).

**Table 1:** Women’s characteristics by survey modality in Lusaka and Nairobi*

	Lusaka			Nairobi		
**Characteristics**	**Phone-based (n = 203)**	**Facility based (n = 233)**	***P*-value**	**Phone-based (n = 300)**	**Facility-based (n = 112)**	***P*-value**
Age group in years			0.011†			0.188
*15–19*	6.9 (4.1, 11.3)	14.6 (10.6, 19.8)		5.0 (3.0, 8.1)	9.8 (5.5, 16.9)	
*20–34*	83.3 (77.4, 87.8)	71.7 (65.5, 77.1)		83.3 (78.7, 87.2)	80.4 (71.9, 86.7)	
*35–49*	9.9 (6.4, 14.8)	13.7 (9.9, 18.8)		11.7 (8.5, 15.8)	9.8 (5.5, 16.9)	
Education category			0.471			0.254
*None*	2.0 (0.7, 5.1)	3.9 (2.0, 7.3)		7.3 (4.9, 10.9)	4.5 (1.9, 10.3)	
*Primary*	27.1 (21.4, 33.6)	24.9 (19.7, 30.9)		22.7 (18.3, 27.8)	29.5 (21.7, 38.6)	
*Secondary and more*	70.9 (64.3, 76.8)	71.2 (65.1, 76.7)		70.0 (64.6, 74.9)	66.1 (56.8, 74.3)	
Marital status			0.576			0.029†
*In union/married*	80.3 (74.2, 85.2)	78.1 (72.3, 83.0)		89.3 (85.3, 92.4)	81.2 (72.9, 87.5)	
*Not in union*	19.7 (14.8, 25.8)	21.9 (17.0, 27.7)		10.7 (7.6, 14.7)	18.8 (12.5, 27.1)	
Employment			0.014†			0.374
*Not employed/no income*	51.2 (44.3, 58.1)	63.1 (56.7, 69.1)		65.0 (59.4, 70.2)	58.0 (48.7, 66.8)	
*Employed (public, private, self)*	36.5 (30.1, 43.3)	30.9 (25.3, 37.2)		22.3 (18.0, 27.4)	25.0 (17.8, 33.9)	
*Informal/casual labor*	12.3 (8.4, 17.6)	6.0 (3.6, 9.9)		12.7 (9.3, 17.0)	17.0 (11.1, 25.1)	
Parity			0.049†			0.168
*1*	22.7 (17.4, 29.0)	31.9 (26.2, 38.2)		26.3 (21.6, 31.6)	35.7 (27.4, 45.0)	
*2–3*	55.7 (48.7, 62.4)	44.8 (38.5, 51.3)		61.3 (55.7, 66.7)	54.5 (45.2, 63.5)	
*≥4*	21.7 (16.5, 27.9)	23.3 (18.3, 29.2)		12.3 (9.1, 16.6)	9.8 (5.5, 16.9)	
History of miscarriage/stillbirth			0.605			0.181
*No*	82.3 (76.4, 86.9)	84.1 (78.8, 88.3)		82.0 (77.2, 86.0)	87.5 (80.0, 92.5)	
*Yes*	17.7 (13.1, 23.6)	15.9 (11.7, 21.2)		18.0 (14.0, 22.8)	12.5 (7.5, 20.0)	
History of caesarean section			0.637			0.715
*No*	80.8 (74.8, 85.7)	79.0 (73.2, 83.7)		91.3 (87.6, 94.0)	90.2 (83.1, 94.5)	
*Yes*	19.2 (14.3, 25.2)	21.0 (16.3, 26.8)		8.7 (6.0, 12.4)	9.8 (5.5, 16.9)	
Any pregnancy complications			0.216			0.128
*No*	92.6 (88.1, 95.5)	89.3 (84.6, 92.7)		86.7 (82.3, 90.1)	81.2 (72.9, 87.5)	
*Yes*	6.9 (4.1, 11.3)	10.7 (7.3, 15.4)		13.3 (9.9, 17.7)	17.9 (11.8, 26.1)	
*Don’t know*	0.5 (0.1, 3.4)	0.0		0.0	0.9 (0.1, 6.1)	
Facility type‡			0.345			0.823
*Health centre/other*	44.8 (38.1, 51.8)	49.4 (43.0, 55.8)		47.7 (42.0, 53.3)	46.4 (37.4, 55.7)	
*Hospital*	55.2 (48.2, 61.9)	50.6 (44.2, 57.0)		52.3 (46.7, 58.0)	53.6 (44.3, 62.6)	
Managing authority						0.306
*Public*	100.0 (98.2, 100.0)§	100.0 (98.4, 100.0)§		74.7 (69.4-, 9.3)	81.2 (72.9, 87.5)	
*Private for profit*	0.0 (0.0, 1.8)§	0.0 (0.0, 1.6)§		8.0 (5.4, 11.7)	4.5 (1.9, 10.3)	
*Private non-profit/faith-based*	0.0 (0.0, 1.8)§	0.0 (0.0, 1.6)§		17.3 (13.4, 22.1)	14.3 (8.9, 22.1)	
Decision to deliver in the facility			0.558			0.074
*Own/joint*	83.7 (78.0, 88.2)	83.7 (78.4, 87.9)		87.3 (83.0, 90.7)	80.4 (71.9, 86.7)	
*Partner/family member’s*	15.8 (11.4, 21.5)	16.3 (12.1, 21.6)		12.7 (9.3, 17.0)	19.6 (13.3, 28.1	
Place of ANC			0.262			0.463
*Same as delivery facility*	62.6 (55.7, 69.0)	63.1 (56.7, 69.1)		34.3 (29.2, 39.9)	30.4 (22.5, 39.5)	
*Other health facility*	34.5 (28.2, 41.3)	36.1 (30.1, 42.4)		65.0 (59.4, 70.2)	67.9 (58.6, 75.9)	
*Home/no ANC*	3.0 (1.3, 6.4)	0.9 (0.2, 3.4)		0.7 (0.2, 2.6)	1.8 (0.4, 6.9)	
Number of ANC contacts			0.043†			0.671
*None*	2.5 (1.0, 5.8)	0.4 (0.1, 3.0)		0.7 (0.2, 2.6)	1.8 (0.4, 6.9)	
*<4*	28.1 (22.3, 34.7)	31.8 (26.1, 38.0)		33.7 (28.5, 39.2)	29.5 (21.7, 38.6)	
*4–7*	60.1 (53.2, 66.6)	63.5 (57.1, 69.5)		61.7 (56.0, 67.0)	66.1 (56.8, 74.3)	
*≥8*	9.4 (6.0, 14.2)	4.3 (2.3, 7.8)		3.7 (2.0, 6.5)	2.7 (0.9, 8.0)	
Assistance during delivery			0.234			0.933
*Physician/specialist*	7.4 (4.5, 11.9)	12.4 (8.8, 17.4)		42.7 (37.2, 48.4)	44.6 (35.7, 54.0)	
*Midwife, TBA, nurse*	89.7 (84.6, 93.2)	82.8 (77.4, 87.2)		55.3 (49.6, 60.9)	53.6 (44.3, 62.6)	
*Other/unskilled*	1.0 (0.2, 3.9)	1.3 (0.4, 3.9)		2.0 (0.9, 4.4)	1.8 (0.4, 6.9)	
*Don’t know*	2.0 (0.7, 5.1)	3.4 (1.7, 6.7)				
Any complications during labor/delivery			0.518			0.617
*No*	88.7 (83.5, 92.4)	90.6 (86.1, 93.7)		91.3 (87.6, 94.0)	92.9 (86.3, 96.4)	
*Yes*	11.3 (7.6, 16.5)	9.4 (6.3, 13.9)		8.7 (6.0, 12.4)	7.1 (3.6, 13.7)	
Private bed for labor/delivery			0.122			0.162
*No*	17.2 (12.6, 23.1)	12.0 (8.4, 16.9)		13.0 (9.6, 17.3)	8.0 (4.2, 14.8)	
*Yes*	82.8 (76.9, 87.4)	88.0 (83.1, 91.6)		87.0 (82.7, 90.4)	92.0 (85.2, 95.8)	
Accompanied by partner/family during labor/delivery			0.604			0.367
*No*	98.5 (95.5, 99.5)	97.9 (94.9, 99.1)		95.3 (92.3, 97.2)	97.3 (92.0, 99.1)	
*Yes*	1.5 (0.5, 4.5)	2.1 (0.9, 5.1)		4.7 (2.8, 7.7)	2.7 (0.9, 8.0)	
Length of stay in facility in hours			0.028†			0.234
*<24*	78.8 (72.6, 83.9)	69.5 (63.3, 75.1)		17.3 (13.4, 22.1)	12.5 (7.5, 20.0)	
*≥24*	21.2 (16.1, 27.4)	30.5 (24.9, 36.7)		82.7 (77.9, 86.6)	87.5 (80.0, 92.5)	
Maternal PNC check before discharge			0.798			0.735
*No*	4.9 (2.7, 8.9)	5.6 (3.3, 9.4)		4.7 (2.8, 7.7)	3.6 (1.3, 9.2)	
*Yes*	93.6 (89.3, 96.3)	93.6 (89.6, 96.1)		95.0 (91.9, 97.0)	96.4 (90.8, 98.7)	
Newborn PNC check before discharge			0.281			0.009†
*No*	6.4 (3.7, 10.7)	9.0 (5.9, 13.4)		7.7 (5.1, 11.3)	0.9 (0.1, 6.1)	
*Yes*	93.1 (88.7, 95.9	89.3 (84.6, 92.7)		92.3 (88.7, 94.9)	98.2 (93.1, 99.6)	
Newborn PNC appointment before discharge			0.013†			0.242
*No*	1.0 (0.2, 3.9)	5.6 (3.3, 9.4)		3.7 (2.0, 6.5)	4.5 (1.9, 10.3)	
*Yes*	99.0 (96.1, 99.8)	93.6 (89.6, 96.1)		96.3 (93.5, 98.0)	94.6 (88.6, 97.6)	
Generally satisfied with care received during childbirth			0.861			0.401
*No*	15.3 (10.9, 20.9)	15.9 (11.7, 21.2)		5.0 (3.0, 8.1)	2.7 (0.9, 8.0)	
*Yes*	84.7 (79.1, 89.1)	84.1 (78.8, 88.3)		94.3 (91.1, 96.5)	97.3 (92.0, 99.1)	
Would recommend the facility to friends/family			0.729			0.612
*No*	10.8 (7.2, 15.9)	13.3 (9.5, 18.3)		4.3 (2.5, 7.3)	2.7 (0.9, 8.0)	
*Yes*	86.7 (81.3, 90.7)	84.1 (78.8, 88.3)		95.3 (92.3, 97.2)	97.3 (92.0, 99.1)	
*Don’t know*	2.5 (1.0, 5.8)	2.6 (1.2, 5.6)		0.3 (0.0, 2.3)		

ANC – antenatal care, PNC – postnatal care, TBA – trained birth attendant

*Presented as % (95% CI) unless specified otherwise.

†*P* < 0.05.

‡Facility type was defined per the health care system in each city as follows. Lusaka – health centre included urban health posts, health centres or clinics. Hospital included first and second level hospitals. Nairobi – health centre included facility levels two and three. Hospital included facility levels four and five.

§Presented as one-sided 97.5% CI.

### Self-reported PCMC by survey modality

**Figure 1:**
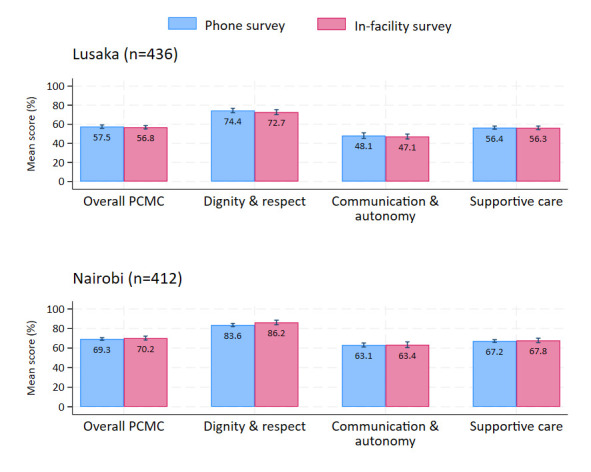
Overall and domain PCMC scores by survey modality in Nairobi and Lusaka.

In Nairobi, the overall self-reported PCMC score was 69.3% (95% CI = 67.9, 70.6) among women surveyed on the phone, compared to 70.2% (95% CI = 68.2, 72.2) among those surveyed in the facility. In Lusaka it was 57.5% (95% CI = 55.7, 59.3) compared to 56.8% (95% CI = 55.0, 58.6) in the phone and facility-based samples respectively. We found no statistically significant difference in overall PCMC scores between the phone and facility-based samples, in both cities. Similarly, there were no statistically significant differences in a given PCMC domain score between groups, in each city (**Figure 1**; Figure S2, Table S3 in the **Online Supplementary Document**).

The crude multilevel models of overall PCMC scores (unscaled) on survey modality indicated that women who participated in exit surveys in-facility reported a higher overall PCMC score by 1.23 points (95% CI = –0.76, 3.22) in Nairobi and 0.19 points (95% CI = –1.92, 2.29) in Lusaka out of 90 points, compared to those surveyed on the phone. This corresponded to an increase of 1.37 pp and 0.21 pp respectively, and was not statistically significant. After adjusting for the relevant differences in women’s characteristics between groups in each city, the results remained consistent. In Nairobi, the effect magnitude was attenuated to 0.75 points (95% CI = –1.21, 2.72) out of 90, or a 0.83 pp higher score in the facility-based sample, whereas in Lusaka it was increased to 0.44 points out of 90 (95% CI = –1.71, 2.60) or 0.49 pp, though neither achieving statistical significance at *P* < 0.05 (**Table 2**). The slight increase in Lusaka’s estimate in the adjusted model is explained by controlling for employment, as women formally employed reported higher PCMC, and there was a larger proportion of formally employed women in the phone sample, thus shifting the overall effect away from the null when controlling for this variable.

**Table 2:** Differences in PCMC scores by survey modality in Lusaka and Nairobi

	Lusaka*				Nairobi†			
**Survey modality: facility-based (in-person‡**	**Crude model, coefficient (95% CI)**	***P*-value**	**Adjusted model, coefficient (95% CI)**	***P*-value**	**Crude model, coefficient (95% CI)**	***P*-value**	**Adjusted model, coefficient (95% CI)**	***P*-value**
Overall PCMC (out of 90 points)	0.19 (–1.92, 2.29)	0.862	0.44 (–1.71, 2.60)	0.689	1.23 (–0.76, 3.22)	0.226	0.75 (–1.21, 2.72)	0.453
Dignity and respect (out of 18 points)	–0.20 (–0.81, 0.42)	0.535	–0.21 (–0.85, 0.42)	0.507	0.56 (0.02, 1.09)§	0.040	0.42 (–0.12, 0.95)	0.127
Communication and autonomy (out of 27 points)	0.08 (–0.90, 1.06)	0.868	0.32 (–0.69, 1.32)	0.537	0.09 (–0.87, 1.06)	0.849	–0.10 (–1.05, 0.86)	0.841
Supportive care (out of 45 points)	0.26 (–0.78, 1.31)	0.621	0.30 (–0.76, 1.35)	0.581	0.54 (–0.43, 1.50)	0.275	0.38 (–0.59, 1.35)	0.439
Observations (n)	436		433		412		410	

CI – confidence interval, PCMC – person-centred maternity care

*Lusaka model adjusted by women’s: age, employment, parity, number of ANC, private bed for labour/delivery, length of stay in facility, newborn PNC appointment received prior to discharge.

†Nairobi model adjusted by women’s: age, marital status, parity, history of miscarriage/stillbirth, pregnancy complications, decision to deliver in facility, private bed for labour/delivery, newborn PNC check prior to discharge.

‡Phone-based as reference.

§Significant at *P* < 0.05.

Zooming in further on differences in women’s reporting of PCMC domains by survey modality, in Lusaka, women surveyed in the facility reported better communication and supportive care, but poorer dignity and respect compared to those surveyed on the phone (**Table 2**). The estimates’ magnitudes were negligible and not statistically significant in crude and adjusted models. In Nairobi, the crude model suggested that women surveyed in the facility reported statistically significantly higher dignity and respect by 0.56 points (out of 18) corresponding to a 3.11 pp increase (*P* < 0.05) compared to those surveyed on the phone. However, the difference was no longer statistically significant in the adjusted model (*P* = 0.127). Women in the facility reported better supportive care by 0.38 points (out of 45) or 0.84 pp, but poorer communication by –0.10 points (out of 27) or –0.37 pp, though neither achieving statistical significance (**Table 2**).

The supplementary subgroup analysis comparing PCMC scores by women’s characteristics between samples in each city suggested no differences in PCMC across survey modalities, even within subgroups of women. However, in Lusaka, among women who reported they would not recommend the facility attended, those surveyed in-facility had lower PCMC scores than those surveyed on the phone (Table S2 in the **Online Supplementary Document**).

## DISCUSSION

Our analysis comparing self-reported PCMC scores between women surveyed in-facility and on mobile phones in Lusaka and Nairobi indicated that women reported on their childbirth care experiences similarly across survey modalities. Moreover, while women scored highest on dignity and respect and lowest on communication and autonomy, the domain scores were also similar between facility and phone-based samples, in each city.

There were some differences in the sample composition between groups. These differences could mask or exacerbate any effect (or lack thereof) of the survey modality on the self-reported PCMC score, especially if they are associated with differing reporting of PCMC. Our multilevel analyses adjusting for such differences in sample composition suggested no statistically significant effect of the survey modality on women’s PCMC scores in both cities. In other words, based on our approach, we found no evidence of courtesy reporting bias in our facility-based exit surveys, contrary to what has been hypothesised [10–13]. Moreover, there was no evidence of courtesy ekreporting bias even within subgroups of women.

Our supplementary analyses, however, pointed to differences in women’s responses to specific questions across survey modalities. For instance, in Nairobi’s phone sample, 16.3% and 2.7% of women reported experiencing verbal abuse and physical abuse at least once respectively, compared to 12.5% and 0% in the facility sample. This difference, although in the direction of courtesy bias, was not statistically significant. The opposite was found in Lusaka, where 6.9% of women in-facility reported experiencing physical abuse at least once, compared to 5.4% of the phone sample; and this difference was statistically significant (Table S4 in the **Online Supplementary Document**). Importantly, these comparisons do not account for inherent differences in the sample composition across groups, which could explain such findings. For instance, there was a larger proportion of adolescent and unemployed women in Lusaka’s facility-based sample, and previous evidence suggests that younger and poorer women are more likely to experience disrespect and abuse during childbirth [20,21].

In rural Kenya, Afulani et al. found that women interviewed in-person in their home reported a statistically significantly lower mean PCMC score than those interviewed in the facility [8]. In a context similar to ours in peri-urban Nairobi, Oluoch-Aridi et al. examined factors associated with PCMC and interviewed women within 6 weeks of delivery, either in the facility, in the community or on the phone. They found that women interviewed on the phone reported significantly lower mean PCMC scores than those interviewed in the facility [13]. Both authors hypothesised that women may have been hesitant to report negative experiences of care while interviewed in the facility [8,13]. Our findings from urban informal settlements in Nairobi and Lusaka were inconsistent with these reports. Our surveys were conducted within one to two weeks of discharge, and the shorter recall compared to other studies could have influenced our findings. Similarly, while models were adjusted by key sociodemographic and facility characteristics in previous studies [8,13], it is also possible that inherent differences in sample compositions remained unadjusted. For instance, our previous analyses suggested that receipt of maternal and newborn PNC before discharge was significantly associated with higher PCMC scores [19]; such provision of care variables were not accounted for in the aforementioned studies.

Other studies on quality perceptions and satisfaction of care comparing women’s responses between in-facility and household surveys in Pakistan and Madagascar found that courtesy bias positively influenced responses in the facility-based sample. Authors recommended restricting in-facility surveys to objective questions least affected by the environment [11,14]. Interestingly, women attending outreach camps for underserved populations in Pakistan were more likely to report negative experiences in the facility, in the opposite direction of courtesy bias, calling for further inquiry [11]. Furthermore, a validation study in northern Nigeria compared facility-based survey responses at discharge to a follow-up phone survey with a subset of women 14 months later, and identified inconsistencies between the two. Authors found poor validity for negative care experiences, with more women in the follow-up phone survey reporting issues such as being denied a birth companion, or a lack of respect for delivery position preferences. However, the comparisons did not account for differences in sample composition. Importantly, the 14-month long recall period was a key limitation, which, along with differing survey modality and location, may explain the reported differences. The authors hypothesized that women’s recall of childbirth care experiences may change over time and across locations [12].

Differences in timing relative to childbirth affect women’s recall of intrapartum and immediate postpartum interventions, which may be influenced by anxiety and fatigue from childbirth [22–24]. In our study, the phone-based survey in Nairobi was administered within one week of facility discharge for most women, and up to two weeks for few women, whereas in-person surveys occurred immediately upon discharge. In Lusaka, both surveys were administered within one to two weeks of discharge. We explored the effect of survey timing on women’s reporting in Nairobi and found that more women in the phone sample, compared to the facility sample, reported experiencing verbal or physical abuse at least once; however, this association was not statistically significant (Table S4 in the **Online Supplementary Document**). We acknowledge that a difference in survey timing may affect women’s recall of their childbirth care experience; for instance, the joy of having a baby might overshadow the reporting of negative experiences immediately after birth. However, our supplementary analyses suggested that the one to two weeks difference did not affect recall significantly. Other studies have measured PCMC with up to six and nine weeks recall postpartum [8,13]; one of them reported significantly lower scores among women surveyed more than one week following discharge [8]. Our study also had differing survey modalities; conducting in-person household visits immediately after discharge would have yielded a stronger comparison, but this approach was more resource-intensive and faced implementation challenges.

The central premise of our study design with varying survey modalities was that women surveyed in the facility would be less likely to accurately report negative experiences of care, as has been found in the literature [8,11,13,14]. While we did not detect significant differences in PCMC scores between mobile phone and facility-based samples, it is crucial to acknowledge that several factors could have influenced our results in either direction. Building rapport and assessing non-verbal cues is more challenging in phone surveys, and participants may be distracted, affecting their engagement. To minimise these factors and inherent differences in data collection techniques between the two approaches, we trained interviewers to maintain rapport over the phone, and gauge attention and understanding of questions. All participants were recruited in-person at the facility, which helped to build rapport earlier on. Additionally, phone surveys began only when the participants confirmed their availability and provided oral consent to continue; interviews were rescheduled if necessary. The goal of our analysis was not to test the phone-based survey technique, but rather to explore whether this approach could alleviate courtesy bias in reporting childbirth care experiences and reduce participant burden. While inherent differences in survey techniques may have affected PCMC estimates positively or negatively, a significantly lower PCMC score in the phone sample would have suggested presence of courtesy bias.

In Nigeria, a qualitative study found that women were highly satisfied with a phone-based approach to evaluate their childbirth care experience, mentioning it made them feel valued and motivated to participate. Women perceived phone surveys as easier, more convenient, and offering better privacy and confidentiality compared to facility-based surveys. Therefore, women appear to be accepting of this approach for assessing childbirth care experiences in settings with adequate mobile phone coverage [25]. Our findings support the benefits of mobile phone surveys for assessing experience of care as they produced comparable results to in-person surveys in the context of urban informal settlements. Nevertheless, further research is needed to address challenges with this approach, including participant engagement, representativeness and biases resulting from inequitable mobile phone coverage and ownership.

Our study has several strengths. To our knowledge, it is the first study specifically exploring courtesy reporting bias in urban informal settlements. The study was implemented in two cities, allowing cross-country comparisons. It also has limitations: our sample was powered to detect PCMC differences between facility and phone samples of 4.2 pp and 3.6 pp in Nairobi and Lusaka, respectively. Hence, it is possible that our sample size was not large enough to detect smaller differences; we aimed to detect a difference that was clinically meaningful and relevant. Similarly, women participating in phone surveys were separate from those who received in-facility surveys. The samples were drawn randomly and independently, and we adjusted for observed differences in sample composition. However, we could not control for unobserved factors such as women’s own expectations and beliefs, which may lead to differential reporting of PCMC by survey modality. These could have obscured any significant differences between groups. Qualitative studies are needed to better understand the influence of such factors on women’s perceived experience of care. The PCMC scale based on women’s self-report is subject to recall bias and may be impacted by survey timing; we limited the recall period to one to two weeks following discharge, which is shorter than other studies [8,13]. Women across facility and phone-based samples may witness social desirability bias in reporting negative experiences; we minimised this through training of data collectors. While mobile-phone surveys may suffer from selection bias resulting from unequal mobile phone coverage, this was not a concern in our urban setting where mobile phone coverage and ownership were high. Lastly, the differences in data collection techniques between in-facility and phone surveys may mask differences in women’s PCMC reporting. Further comparative studies using hybrid data collection approaches are needed to corroborate our findings.

## CONCLUSIONS

To conclude, we found no evidence of courtesy bias in women’s reporting of PCMC in facility-based client exit surveys in urban informal settlements in Nairobi and Lusaka, contrary to previous evidence. However, factors related to differing survey techniques between a phone-based and an in-person facility-based approach may have clouded any possible differences. Our findings suggest that in-person and mobile phone-based approaches produce similar results and could be used to accurately measure PCMC in resource-constrained settings. Previous evidence indicates that women prefer phone-based surveys to report on experiences of childbirth care [25]; this approach could reduce financial costs and challenges of conducting surveys in resource-constrained settings. Further research is needed to better understand the potential effects of mobile phone surveys on women’s reporting accuracy, and to address challenges pertaining to this approach.

## Additional material


Online Supplementary Document
