# Supplementary material for: Assessing courtesy reporting bias in facility-based surveys on person-centred maternity care: evidence from urban informal settlements in Nairobi and Lusaka
Source: J Glob Health. 2025 Mar 28;15:04090. doi: 10.7189/jogh.15.04090 (PMC11949514; doi:10.7189/jogh.15.04090)
Supplement: Online Supplementary Document [file jogh-15-04090-s001.pdf]

Supplement to: Jiwani SS, Mutua M, Jacobs C, Musukuma M, Njeri A, Adero G, Ngosam D,Abajobir A, Faye C, Boerma T, Amouzou A. Assessing courtesy reporting bias in facility-basedsurveys on person-centred maternity care: evidence from urban informal settlements in Nairobiand Lusaka. J Glob Health. 2025;15:04090.

Supplementary table 1S. PCMC scale questions, response categories and scoring

Question	Response categories	PCMC scoring (out of 90)
**DIGNITY & RESPECT**		
Did the doctors, nurses, or other staff at the facility treat you with respect?	No, never	0
	Yes, a few times	1
	Yes, most of the time	2
	Yes, all the time	3
Did the doctors, nurses, or other staff at the facility treat you in a friendly manner?	No, never	0
	Yes, a few times	1
	Yes, most of the time	2
	Yes, all the time	3
Did you feel that they shouted at you, scolded, insulted, threatened, or talked to you rudely?	No, never	3
	Yes, once	2
	Yes, a few times	1
	Yes, many times	0
	Refused to respond	3
Did you feel like you were treated roughly like pushed, beaten, slapped, pinched, physically restrained, or gagged?	No, never	3
	Yes, once	2
	Yes, a few times	1
	Yes, many times	0
	Refused to respond	3
During examinations in the labor room, were you covered up with a cloth or blanket, or screened with a curtain so that you did not feel exposed?	No, never	0
	Yes, a few times	1
	Yes, most of the time	2
	Yes, all the time	3
Do you feel like your health information was or will be kept confidential at this facility?	No, never	0
	Yes, a few times	1
	Yes, most of the time	2

	Yes, all the time	3
**COMMUNICATION & AUTONOMY**		
During your time in the health facility did the doctors, nurses, or other health-care providers introduce themselves to you when they first came to see you?	No, none of them	0
	Yes, a few of them	1
	Yes, most of them	2
	Yes, all of them	3
Did the doctors, nurses, or other health-care providers call you by your name?	No, never	0
	Yes, a few times	1
	Yes, most of the time	2
	Yes, all the time	3
Did you feel like the doctors, nurses or other staff at the facility involved you in decisions about your care?	No, never	0
	Yes, a few times	1
	Yes, most of the time	2
	Yes, all the time	3
	Did not have to make any decisions	3
During the delivery, do you feel like you were able to be in the position of your choice?	No, never	0
	Yes, for a short time	1
	Yes, most of the time	2
	Yes, all the time	3
Did the doctors, nurses, or other staff at the facility speak to you in a language you could understand?	No, never	0
	Yes, a few times	1
	Yes, most of the time	2
	Yes, all the time	3
Did the doctors, nurses, or other staff at the facility ask your permission or consent before doing procedures on you?	No, never	0
	Yes, a few times	1
	Yes, most of the time	2
	Yes, all the time	3
Did the doctors, nurses, or other staff at the facility explain to you why they were doing examinations or procedures on you?	No, never	0
	Yes, a few times	1
	Yes, most of the time	2

	Yes, all the time	3
Did the doctors, nurses, or other staff at the facility explain to you why they were giving you any medicine?	No, never	0
	Yes, a few times	1
	Yes, most of the time	2
	Yes, all the time	3
	Did not get any medicine	3
Did you feel you could ask the doctors, nurses, or other staff at the facility any questions you had?	No, never	0
	Yes, a few times	1
	Yes, most of the time	2
	Yes, all the time	3
**SUPPORTIVE CARE**		
How did you feel about the amount of time you waited to receive care? Would you say it was:	Very short	3
	Somewhat short	2
	Somewhat long	1
	Very long	0
Did the doctors and nurses at the facility show concern for your feelings about your delivery?	No, never	0
	Yes, a few times	1
	Yes, most of the time	2
	Yes, all the time	3
Did the doctors, nurses, or other staff at the facility try to understand your anxieties?	No, never	0
	Yes, a few times	1
	Yes, most of the time	2
	Yes, all the time	3
	Did not have any anxiety	3
When you needed help, did you feel the doctors, nurses, or other staff at the facility paid attention?	No, never	0
	Yes, a few times	1
	Yes, most of the time	2
	Yes, all the time	3
Do you feel the doctors or nurses did everything they could to help control your pain?	No, never	0
	Yes, a few times	1
	Yes, most of the time	2

	Yes, all the time	3
Were you allowed to have someone you wanted (outside of staff at the facility, such as family or friends) to stay with you during labor?	No, never	0
	Yes, a few times	1
	Yes, most of the time	2
	Yes, all the time	3
	I did not want anyone to stay with me	3
Were you allowed to have someone you wanted to stay with you during delivery?	No, never	0
	Yes, a few times	1
	Yes, most of the time	2
	Yes, all the time	3
	I did not want anyone to stay with me	3
Did you feel the doctors, nurses, or other staff at the facility took good care of you, at the best of their ability?	No, never	0
	Yes, a few times	1
	Yes, most of the time	2
	Yes, all the time	3
Did you feel you could completely trust the doctors, nurses, or other staff at the facility with regards to your care?	No, never	0
	Yes, a few times	1
	Yes, most of the time	2
	Yes, all the time	3
Do you think there were enough health staff in the facility to care for you?	No, never	0
	Yes, a few times	1
	Yes, most of the time	2
	Yes, all the time	3
Thinking about the labor and postnatal wards, did you feel the health facility was crowded?	No, never	3
	Yes, a few times	2
	Yes, most of the time	1
	Yes, all the time	0
Thinking about the wards, washrooms, and the general environment of the health facility, would you	Very dirty	0
	Dirty	1

say the facility was very clean, clean, dirty, or very dirty?	Clean	2
	Very clean	3
Was there running water in the facility?	No, never	0
	Yes, a few times	1
	Yes, most of the time	2
	Yes, all the time	3
Was there electricity in the facility?	No, never	0
	Yes, a few times	1
	Yes, most of the time	2
	Yes, all the time	3
In general, did you feel safe in the health facility?	No, never	0
	Yes, a few times	1
	Yes, most of the time	2
	Yes, all the time	3

Supplementary figure 1S. Normality of PCMC outcome

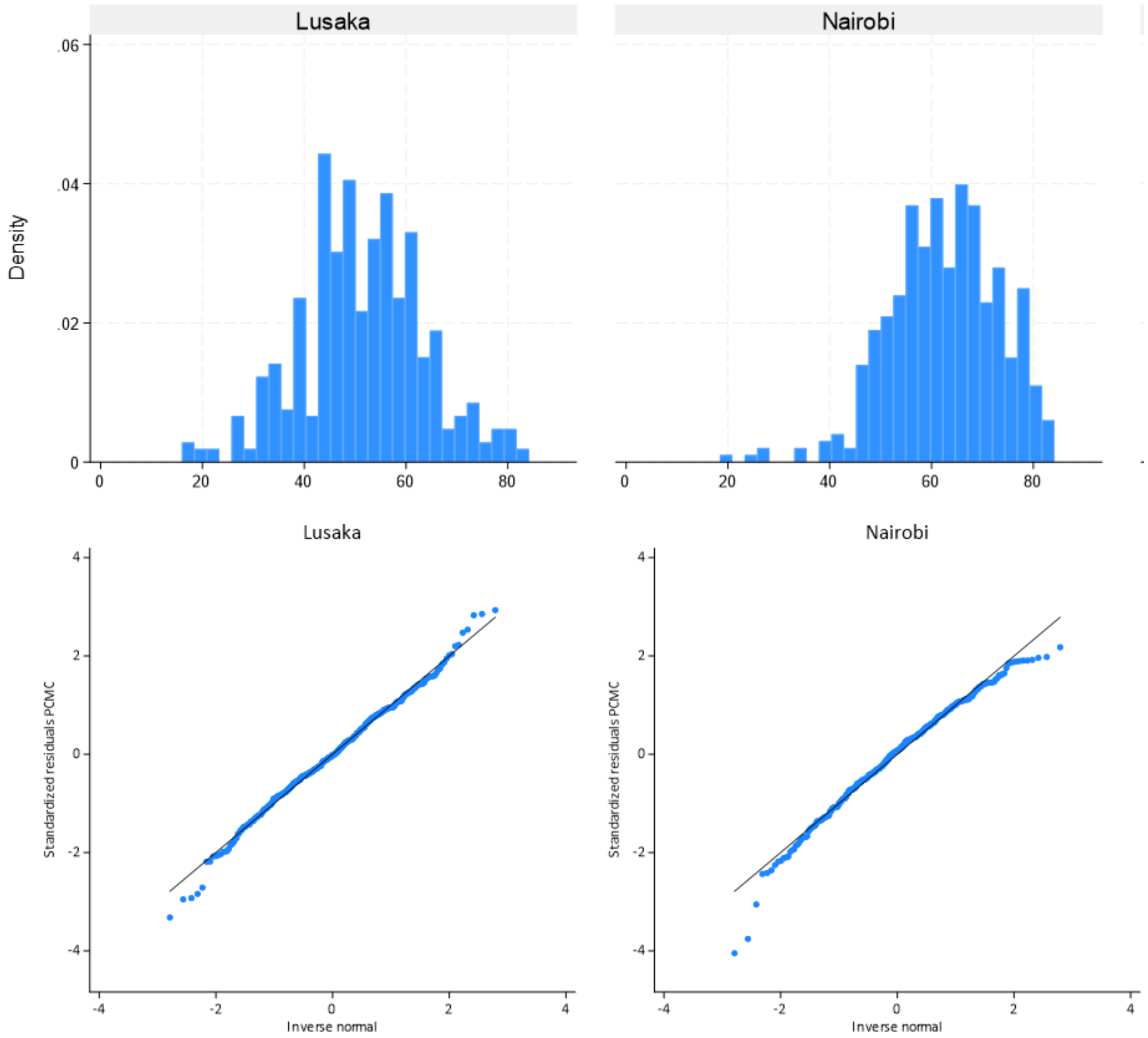

Supplementary table 2S. PCMC scores (%) by women's socio-demographic characteristics and survey modality.

	LUSAKA				NAIROBI			
	Phone-based (n=203)		Facility-based (n=233)		Phone-based (n=300)		Facility-based (n=112)	
**Socio-demographic characteristics**	Mean	(95% CI)	Mean	(95% CI)	Mean	95% CI	Mean	95% CI
**Age group**								
15-19 yrs	55.7	(49.3 - 62.1)	53.7	(49.7 - 57.7)	70.7	(64.9 - 76.5)	70.9	(63.9 - 77.9)
20-34 yrs	57.7	(55.7 - 59.6)	57.5	(55.3 - 59.7)	69.2	(67.7 - 70.8)	69.3	(67.2 - 71.5)
35-49 yrs	57.8	(51.9 - 63.6)	56.4	(52.0 - 60.8)	68.9	(65.7 - 72.1)	76.3	(69.3 - 83.2)
**Education category**								
None	44.2	(36.3 - 52.1)	53.1	(49.6 - 56.6)	65.3	(60.4 - 70.2)	70.0	(58.5 - 81.5)
Primary	58.1	(54.8 - 61.4)	57.7	(54.2 - 61.1)	72.0	(69.5 - 74.4)	72.1	(68.0 - 76.1)
Secondary +	57.7	(55.6 - 59.8)	56.7	(54.5 - 58.9)	68.8	(67.1 - 70.5)	69.3	(67.0 - 71.7)
**Marital status**								
In union	57.4	(55.4 - 59.4)	56.8	(54.7 - 58.8)	69.7	(68.3 - 71.1)	71.0	(68.8 - 73.2)
Not in union	58.1	(54.2 - 62.0)	56.9	(53.6 - 60.2)	65.6	(60.4 - 70.7)	66.6	(61.9 - 71.3)
**Employment**								
Not employed/no income	56.9	(54.5 - 59.4)	55.1	(53.1 - 57.2)	69.8	(68.1 - 71.5)	69.5	(67.2 - 71.8)
Employed (public, private, self)	59.3	(56.4 - 62.3)	59.3	(55.9 - 62.7)	69.1	(66.2 - 72.1)	70.3	(65.7 - 74.9)
Informal/casual labor	54.7	(49.8 - 59.6)	61.1	(52.7 - 69.5)	66.8	(63.2 - 70.4)	72.2	(66.5 - 77.9)
**Parity**								
1 child	58.0	(53.9 - 62.1)	54.5	(51.3 - 57.8)	67.9	(65.7 - 70.1)	68.7	(65.7 - 71.7)
2 to 3 children	57.0	(54.8 - 59.1)	58.8	(56.3 - 61.4)	69.5	(67.6 - 71.3)	71.1	(68.2 - 73.9)
4+ children	58.5	(54.2 - 62.8)	56.1	(52.5 - 59.7)	71.2	(67.4 - 75.0)	70.5	(63.2 - 77.8)
**Facility type***								
Health center/other	60.1	(57.3 - 62.9)	55.6	(53.3 - 58.0)	75.6	(74.0 - 77.3)	74.2	(71.3 - 77.1)
Hospital	55.4	(53.2 - 57.7)	57.9	(55.3 - 60.5)	63.5	(61.8 - 65.2)	66.7	(64.2 - 69.2)
**Managing authority**								
Public					66.9	(65.3 - 68.5)	68.9	(66.7 71.1)
Private: for profit					76.7	(73.3 - 80.2)	74.0	(64.9 83.1)

Private: non-profit/faith-based					76.0	(73.6 - 78.4)	76.2	(71.0 81.3)
**Decision to deliver in facility**								
Own/joint	58.4	(56.5 - 60.3)	56.8	(54.9 - 58.7)	69.7	(68.3 - 71.2)	71.2	(68.9 - 73.5)
partner/family member's	52.8	(48.4 - 57.3)	56.8	(52.1 - 61.5)	66.0	(63.0 - 69.0)	65.9	(62.0 - 69.8)
**Antenatal Care**								
**Number of ANC contacts**								
None	59.3	(48.8 - 69.9)	65.6		60.0	(55.4 - 64.6)	75.0	(52.6 - 97.4)
<4 contacts	56.9	(52.9 - 60.9)	57.1	(53.9 - 60.4)	69.1	(66.6 - 71.7)	70.5	(66.5 - 74.5)
4-7 contacts	58.4	(56.2 - 60.6)	56.4	(54.2 - 58.6)	69.5	(67.9 - 71.1)	69.4	(67.1 - 71.7)
8+ contacts	53.1	(50.3 - 55.9)	59.7	(52.4 - 66.9)	68.8	(58.3 - 79.2)	83.7	(77.2 - 90.3)
**Childbirth care**								
**Assistance during delivery**								
Physician/specialist	58.2	(51.2 - 65.2)	60.5	(55.6 - 65.4)	73.1	(70.8 - 75.3)	73.8	(70.8 - 76.9)
Midwife, TBA, nurse	57.4	(55.5 - 59.3)	56.6	(54.7 - 58.5)	66.3	(64.6 - 67.9)	67.5	(65.0 - 70.0)
Other/unskilled**	71.7	(70.9 - 72.4)	48.9	(44.8 - 53.0)				
Don't Know**	52.2	(48.1 - 56.3)	51.0	(38.9 - 63.0)	71.3	(68.2 - 74.4)	60.0	(52.3 - 67.7)
**Private bed for labor/delivery**								
No	57.8	(54.1 - 61.4)	48.8	(43.3 - 54.4)	63.0	(59.7 - 66.4)	61.4	(56.9 - 65.8)
Yes	57.5	(55.5 - 59.5)	57.9	(56.1 - 59.7)	70.2	(68.7 - 71.6)	70.9	(68.8 - 73.0)
**Accompanied by partner/family during labor/delivery**								
No	57.5	(55.7 - 59.3)	56.7	(54.9 - 58.5)	69.1	(67.7 - 70.5)	69.8	(67.8 - 71.8)
Yes	62.6	(46.8 - 78.3)	59.6	(46.8 - 72.3)	72.9	(67.4 - 78.4)	83.3	(73.5 - 93.2)
**Length of stay in facility**								
<24h	57.7	(55.6 - 59.7)	55.9	(53.7 - 58.0)	71.8	(68.1 - 75.5)	73.3	(67.1 - 79.6)
≥24h	57.0	(53.4 - 60.6)	58.9	(55.8 - 62.0)	68.7	(67.3 - 70.2)	69.7	(67.6 - 71.8)
**Maternal PNC check before discharge**								
No	43.3	(36.5 - 50.1)	43.0	(39.7 - 46.3)	56.8	(46.6 - 67.1)	68.6	(60.8 - 76.4)
Yes	58.2	(56.4 - 60.0)	57.5	(55.6 - 59.3)	69.9	(68.6 - 71.2)	70.2	(68.2 - 72.3)
**Newborn PNC check before discharge**								

No	43.1	(37.1	-	49.1)	47.0	(42.7	-	51.3)	57.3	(52.1	-	62.5)				
Yes	58.5	(56.7	-	60.2)	57.9	(56.0	-	59.7)	70.3	(68.9	-	71.6)	70.3	(68.3	-	72.3)
<u>**Satisfaction of childbirth care**</u>																
**Generally satisfied with care received during childbirth**																
No	46.2	(42.4	-	50.0)	39.6	(35.7	-	43.4)	47.8	(41.6	-	54.0)	64.1	(53.7	-	74.4)
Yes	59.6	(57.7	-	61.4)	60.0	(58.4	-	61.7)	70.5	(69.2	-	71.8)	70.3	(68.3	-	72.4)
**Would recommend facility to friends/family**																
No	46.8	(41.7	-	51.9)	37.7	(34.0	-	41.4)	47.3	(40.5	-	54.1)	63.0	(51.9	-	74.0)
Yes	59.1	(57.2	-	60.9)	59.9	(58.3	-	61.6)	70.4	(69.2	-	71.7)	70.4	(68.3	-	72.4)

** Facility type was defined per the healthcare system in each city as follows:*
Lusaka: Health center included urban health posts, health centers or clinics; hospital included first and second level hospitals.
Nairobi: Health center included facility levels 2 & 3; Hospital included facility levels 4 & 5.
***Small sample sizes (n<10).*

<u>Supplementary table 3S. Mean PCMC scores (%) by survey modality.</u>

	Lusaka			Nairobi		
	Phone (n=203)	Facility (n=233)	p value	Phone (n=300)	Facility (n=112)	p value
**Overall PCMC score** % (95% CI)	57.5% (55.7, 59.3)	56.8% (55.0, 58.6)	0.568	69.3% (67.9, 70.6)	70.2% (68.2, 72.2)	0.483
**Dignity & respect score** % (95% CI)	74.4% (72.1, 76.7)	72.7% (70.1, 75.3)	0.338	83.6% (81.9, 85.2)	86.2% (83.9, 88.5)	0.065
**Communication & autonomy score** % (95% CI)	48.1% (45.2, 51.0)	47.1% (44.5, 49.7)	0.611	63.1% (61.1, 65.2)	63.4% (60.5, 66.3)	0.893
**Supportive care score** % (95% CI)	56.4% (54.8, 58.1)	56.3% (54.4, 58.1)	0.886	67.2% (65.8, 68.6)	67.8% (65.6, 70.1)	0.653

Supplementary figure 2S. Distribution of PCMC scores (%) by survey modality in Lusaka and Nairobi

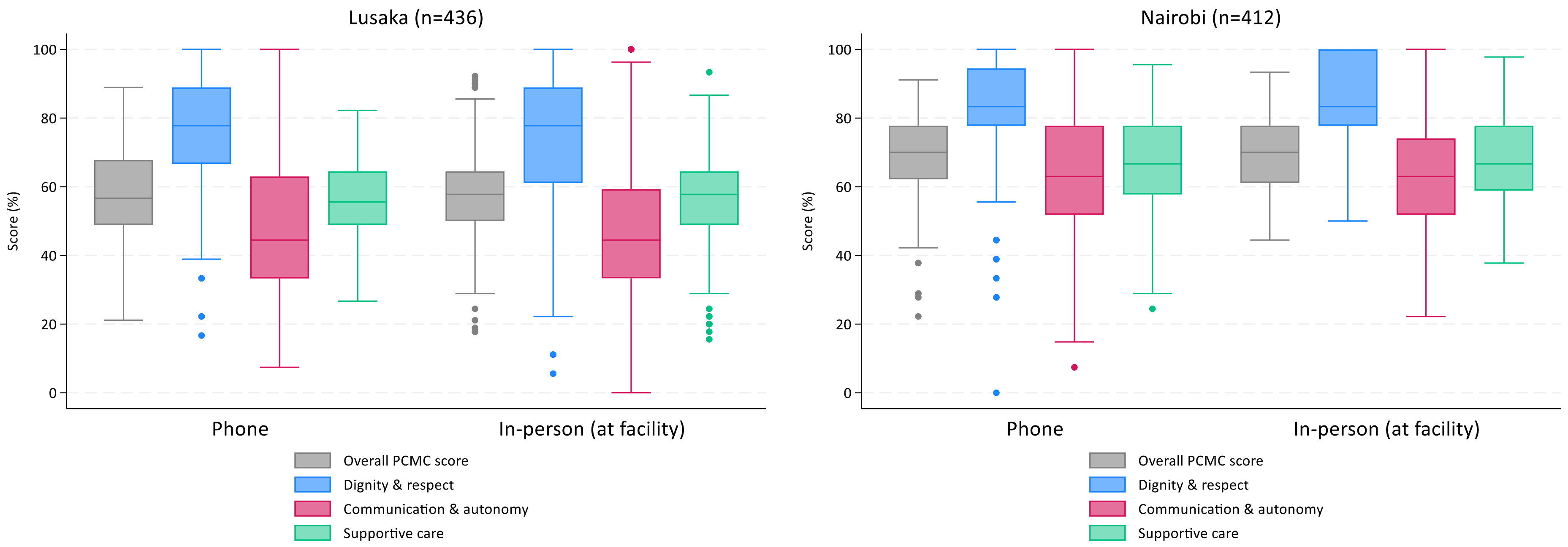

Supplementary table 4S. Responses to dignity and respect PCMC questions by survey modality

		LUSAKA			NAIROBI		
Question	Response categories	Phone-based (%)	In-Facility (%)	Chi2 p value	Phone-based (%)	In-Facility (%)	Chi2 p value
DIGNITY & RESPECT							
Did the doctors, nurses, or other staff at the facility treat you with respect?	No, never	3.0	6.0	0.169	1.3	0.0	0.637
	Yes, a few times	16.7	15.9		3.3	2.7	
	Yes, most of the time	37.9	30.0		27.7	29.5	
	Yes, all the time	42.4	48.1		67.7	67.9	
Did the doctors, nurses, or other staff at the facility treat you in a friendly manner?	No, never	3.4	7.7	0.104	3.3	1.8	0.829
	Yes, a few times	21.2	15.9		6.3	6.2	
	Yes, most of the time	32.5	28.8		26.0	28.6	
	Yes, all the time	42.9	47.6		64.3	63.4	
Did you feel that they shouted at you, scolded, insulted, threatened, or talked to you rudely?	Yes, many times	4.9	6.9	0.447	1.3	0.0	0.112
	Yes, a few times	6.9	10.3		6.0	1.8	
	Yes, once	8.9	7.3		9.0	10.7	
	No, never	79.3	75.5		83.7	86.6	
	Refused to respond	0.0	0.0		0.0	0.9	
Did you feel like you were treated roughly like pushed, beaten, slapped, pinched, physically restrained, or gagged?	Yes, many times	0.5	1.3	**0.044**	0.7	0.0	0.385
	Yes, a few times	1	4.3		1.0	0.0	
	Yes, once	3.9	1.3		1.0	0.0	
	No, never	94.6	93.1		97.3	100.0	
	Refused to respond	0.0	0.0		0.0	0.0	
During examinations in the labor room, were you covered up with a cloth or blanket, or screened with a curtain so that you did not feel exposed?	No, never	27.1	35.6	**0.008**	25.7	19.6	0.149
	Yes, a few times	22.2	10.7		9.7	15.2	
	Yes, most of the time	19.7	19.3		25.3	19.6	

	Yes, all the time	31	34.3		39.3	45.5	
Do you feel like your health information was or will be kept confidential at this facility?	No, never	5.4	11.6	**0.030**	3.7	1.8	0.460
	Yes, a few times	17.7	21		6.3	4.5	
	Yes, most of the time	53.2	41.2		33.3	29.5	
	Yes, all the time	23.6	26.2		56.7	64.3	
